# Topographic mapping of the interfaces between human and aquatic mosquito habitats to enable barrier targeting of interventions against malaria vectors

**DOI:** 10.1098/rsos.161055

**Published:** 2018-05-23

**Authors:** Victoria M. Mwakalinga, Benn K. D. Sartorius, Alex J. Limwagu, Yeromin P. Mlacha, Daniel F. Msellemu, Prosper P. Chaki, Nicodem J. Govella, Maureen Coetzee, Stefan Dongus, Gerry F. Killeen

**Affiliations:** 1 School of Urban and Regional Planning, Department of Housing and Infrastructure Planning, Ardhi University, PO Box 35176, Dar es Salaam, Tanzania; 2 Department of Environmental Health and Ecological Sciences, Ifakara Health Institute, Kiko Avenue, Mikocheni, PO Box 78373, Dar es Salaam, Tanzania; 3 School of Public Health, Faculty of Health Sciences, University of the Witwatersrand, Johannesburg, South Africa; 4 Wits Research Institute for Malaria and Wits/MRC Collaborating Centre for Multidisciplinary Research on Malaria, School of Pathology, University of the Witwatersrand, Johannesburg, South Africa; 5 Discipline of Public Health Medicine, School of Nursing and Public Health, University of KwaZulu-Natal, Durban, South Africa; 6 Department of Epidemiology and Public Health, Swiss Tropical and Public Health Institute, Socinstrasse 57, PO Box, 4002 Basel, Switzerland; 7 Vector Biology Department, Liverpool School of Tropical Medicine, Pembroke Place, Liverpool L3 5QA, UK

**Keywords:** geophysical topography, spatial modelling, *Plasmodium falciparum*, malaria, *Anopheles gambiae*, barrier-targeted interventions

## Abstract

Geophysical topographic metrics of local water accumulation potential are freely available and have long been known as high-resolution predictors of where aquatic habitats for immature *Anopheles* mosquitoes are most abundant, resulting in elevated densities of adult malaria vectors and human infection burden. Using existing entomological and epidemiological survey data, here we illustrate how topography can also be used to map out the interfaces between wet, unoccupied valleys and dry, densely populated uplands, where malaria vector densities and infection risk are focally exacerbated. These topographically identifiable geophysical boundaries experience disproportionately high vector densities and malaria transmission risk, because this is where *Anopheles* mosquitoes first encounter humans when they search for blood after emerging or ovipositing in the valleys. Geophysical topographic indicators accounted for 67% of variance for vector density but for only 43% for infection prevalence, so they could enable very selective targeting of interventions against the former but not the latter (targeting ratios of 5.7 versus 1.5 to 1, respectively). So, in addition to being useful for targeting larval source management to wet valleys, geophysical topographic indicators may also be used to selectively target adult *Anopheles* mosquitoes with insecticidal residual sprays, fencing, vapour emanators or space sprays to barrier areas along their fringes.

## 1. Introduction

Recent attempts to demonstrate the value of targeting malaria transmission *hotspots* with elevated vector densities and human infection burden yielded disappointing results in a rural setting with dispersed settlement patterns and variable but ubiquitous transmission [1]. However, it has been suggested that this geographically selective approach might be more effective in settings with more aggregated populations, because this will result in less dispersal of *Anopheles* mosquitoes and dispersion of malaria transmission across the landscape [1]. Taking this rationale further, the urban contexts of towns and cities may perhaps offer the most ideal settings for geographical targeting of supplementary interventions: dense human populations surrounding aquatic larval habitats allow *Anopheles* mosquitoes to feed nearby and then return to oviposit, thus limiting their dispersal and the diffusion of malaria transmission across the landscape [2–4]. Also, urban settings have lower transmission intensity than rural areas, because high population density dilutes out vector biting burden [5,6] and urban planning can reduce it even further [2,7,8]. Transmission in urban settings may therefore be more vulnerable to control with effective interventions generally and targeted interventions specifically. Furthermore, infrastructure, institutional capacity and governance systems are often better developed than in rural areas, and greater numbers of people can be protected per unit of surface area covered, so several intervention strategies may be viable in towns and cities that would otherwise be considered infeasible [2,8–11]. However, one of the greatest challenges to selective geographical targeting of malaria is the very fine scales that heterogeneity occurs at [12–16]. Hotspots can occur at scales of less than 100 m, and even at the level of single households [1,13,14], so mapping these out at sufficiently high resolution may not be realistically feasible across large programmatic scales with existing entomological and epidemiological survey techniques [1,14,17].

Geophysical topographic indicators of local water accumulation potential have long been recognized as high-resolution (as fine as 10–20 m) predictors of locations with abundant aquatic larval habitats for mosquitoes and therefore high densities of adult vectors and human malaria infection burden [18–25]. Geophysical topographic predictors of local wetness could therefore be useful for identifying even very small geographical hotspots of malaria transmission. Specifically, topographic predictors of local wetness could enable spatial targeting of supplementary interventions at the very fine spatial resolutions that are probably required to achieve improvements in impact or efficiency, relative to blanket coverage [1,14,17].

Dar es Salaam in Tanzania is a typical African coastal city, where local government programmes for larval source management have been highly effective [26] and sustainably institutionalized [27]. At the time of this study, three-quarters of all malaria vector mosquitoes and half of all human infections occur in small, scattered, haphazardly distributed loci, outside of spatially aggregated hotspots that could be detected with existing field survey techniques [17]. This study was therefore undertaken to assess a comprehensive range of geophysical topographic indicators as high-resolution predictors of malaria transmission, with which to map out areas for targeting larval source management interventions. However, in addition to the expected hydrological indicators of where larval habitat occurred, novel geophysical topographic indicators for barrier targeting of supplementary vector control interventions against adult *Anopheles* mosquitoes were also identified.

## 2. Material and methods

### 2.1. Study area

The study was conducted in urban and peri-urban areas of Dar es Salaam, covering a total of 498 km^2^ and encompassing an overall population of 3.6 million people [26]. The city is situated along the shores of the Indian Ocean in Tanzania, having a hot (mean daily temperature of 26°C) and humid climate (two rainy seasons), which provides ideal conditions for *Anopheles gambiae* proliferation and malaria transmission [28]. Urban Dar es Salaam has historically experienced relatively modest, but nevertheless stable and persistent, malaria transmission intensity, with an entomological inoculation rate just above one infectious bite per person per year [26,28].

The geophysical topography of Dar es Salaam is divided into three distinct zones [29]: *the upland zones* (greater than 100 m.a.s.l.), consisting of hilly areas, with plains dissected by many well-drained streams on the western and northern sides of the city; *the middle plateau* (62–100 m.a.s.l.), which is characterized by gently undulating plateaus with isolated hills and rocky outcrops, dissected by many streams in the central parts of the city; *and the lowlands* (0–62 m.a.s.l.), consisting of uniform relief with gentle slopes invariably less than 3%, except along the immediate margins of the three big river valley systems, the Msimbazi River in the central parts of the city, the Keko drain and the Mzinga River in the southern parts. This zone consists of the coastal belt extending from the northern to the southern parts of the city [29].

The study encompassed 71 of the 90 (79%) wards in the city, of which 15 are the same original wards first covered with larvicide by the Dar es Salaam Urban Malaria Control Programme (UMCP) during its operational research phase that ended in 2009 [28]. Following transition to fully programmatic funding and management [26,27], blanket coverage with microbial larvicides had just been reintroduced to these same 15 wards at the outset of this study in March 2010, before comprehensive scale up to cover 56 wards by January 2012 [30]. Between March and October 2010, a coated granule larvicide formulation (VectoBac®; Valent BioSciences Corporation) of *Bacillus thuringiensis* var. *israelensis* (*Bti*) was applied under short-term management of a private sector contractor (Research Triangle International), before the government of Tanzania (GoT) through the Ministry of Health and Social Welfare (MoHSW) took over direct management of all larviciding activities in January 2011. The MoHSW reinitiated larvicide application in February 2011 using the same granule *Bti* product as used by the external contractor until the end of July 2011, before moving to a pre-diluted aqueous suspension formulation (Bactivec®, Labiofam®) of *Bti* from September 2011 through to the end of this study [26].

### 2.2. Data collection and collation

This study uses secondary data collected from two distinct phases of entomological and epidemiological surveys which have been described in detail elsewhere [26]. Briefly, malaria infection prevalence was recorded using two distinct phases of rolling, cross-sectional, cluster-sampled household surveys with rapid diagnostic tests for *Plasmodium falciparum* malaria from March 2010 to July 2012, while a community-based trapping scheme to conduct longitudinal surveys of mosquito densities at high spatial resolution was scaled up across the study area over the same period, with a brief interruption over the last quarter of 2010 and the first of 2011.

Additionally, a comprehensive range of geophysical topographical indicators of hydrology (table 1) were derived from a 20 × 20 m grid of the study area. This was done with SAGA GIS, a free open source software package (www.saga-gis.org) [43], based on a digital elevation model of Dar es Salaam with 20 m resolution, which was freely obtained from the Faculty of Geo-Information Science and Earth Observation, University of Twente, Enschede, The Netherlands. The weighted averages (based on ten cell units (TCUs), which represent the lowest level administrative unit in Tanzania, usually consisting of at least 10 to a maximum of 100 houses) for discrete geophysical topographic variables were computed to obtain values of each geophysical topographic predictor for each of the study locations. The extracted values of geophysical topography were linked with those of mosquito vector densities and infection prevalence in each location using TCU centroids, which were calculated with ArcGIS v.10 software (ESRI Corporation, Redlands, CA). The subsequent analyses were done at the TCU level. The boundaries of both the sampling clusters for mosquitoes and the cross-sectional sampling clusters were obtained through the participatory mapping approach as described elsewhere [30,44]. A handheld global positioning system receiver with an accuracy of 5 m was used to provide spatial references of specific locations for Ifakara tent traps used to catch mosquitoes, as well as households included in cross-sectional surveys. These were the basis for calculating the centroids of the sampling clusters.

**Table 1.** Definitions of geophysical topographical indicators that describe local hydrology in Dar es Salaam city.

variables	descriptions
altitude above channel network	This is the vertical distance to a channel base level. It is the difference between the interpolated channel network base level and this base level from the original elevations [25,31].
aspect	This is the direction of the maximum gradient and relates to the degree of solar exposure. The aspect determines the effect of solar heating, air temperature and moisture (microclimatic influence). The orientation that the hill slope faces ranges from 0° to 360° (0° and 360° north, 90° east, 180° south and 270° west) [22].
channel network	This is a naturally created drain and may be either dry (valley) or conveying water (river). It defines the extent/coverage of rivers and other local drainages/valleys [25,31].
elevation	The vertical distance of a point or level on or affixed to the surface of the Earth, measured from mean sea level. Elevation was derived from the digital elevation model. Primarily influences water movement throughout a landscape and within drainage channels.
hill shading	This shows local areas with shadows thrown upon raised landscapes (http://support.esri.com/en/knowledgebase/GISDictionary/term/hillshading). The higher the shading values, the lighter and warmer the surface (more exposed to sun rays); and the lower the hill shading values, the darker and cooler the surface becomes.
profile curvature	This is the curvature in a horizontal plane and is perpendicular to the direction of the maximum slope. A positive value indicates that the surface is sidewardly convex at that cell. A negative plan indicates that the surface is sidewardly concave at that cell. A value of zero indicates that the surface is linear. Profile curvature relates to the convergence and divergence of flow across a surface [32] (Raster curvature. http://www.et-st.com/et_surface/userguide/Raster/ETG_RasterCurvature.htm).
planform curvature	Profile curvature is the curvature intersecting with the plane defined by the *Z*-axis and maximum gradient direction. Positive values describe convex profile curvature, and negative values describe concave profile. A negative value indicates that the surface is upwardly convex at that cell, and a positive value indicates that the surface is upwardly concave; a value of zero indicates that the surface is linear. The profile curvature affects the flow velocity of water draining the surface and influences erosion and deposition. In locations with convex (negative) (raster curvature. http://www.et-st.com/et_surface/userguide/Raster/ETG_RasterCurvature.htm).
slope	This is a measure of the angle of descent or ascent for each pixel (calculated as the rate of change in altitude). The angle of incline on a hillside is called the slope, and the lower the slope value, the flatter the terrain; the higher the slope value, the steeper the terrain. Slope has a strong influence on overland and subsurface flow velocity, drainage and accumulation of water [33].
topographic convergence index (TCI)	The TCI is the direction of water flow between adjacent cells based on the aspects of neighbouring cells. It determines whether water flow from neighbouring cells diverges (positive values less than or equal to 100 indicating dry areas) or converges (negative values greater than or equal to −100 indicating saturated areas) [25,31].
topographic position index (TPI)	The difference between the elevation at a cell and the average elevation in a neighbourhood surrounding that cell. TPI is used to measure geophysical topographic slope positions and to automate landform classifications whereby negative values indicate valleys and positive values signify ridges [34].
topographic ruggedness index (TRI)	A measure of terrain roughness may be the standard deviation of the slope, the standard deviation of the elevation, the slope convexity, the variability of the plan convexity or some other measure of geophysical topographic texture [34]. In this study, TRI corresponds to the average elevation change between any point on a digital elevation model grid (one grid is 20 × 20 m) and its surrounding area [35].
topographic wetness index (TWI)	This is the estimate of the predicted water accumulation (provides an index of potential moisture availability), and it was calculated as the ratio of the contributing upslope drainage area and the local slope as developed by Tarboton [36]. The TWI ranges from less than 0 to infinity [37]. High values (TWI > 20) are found for converging terrains and valleys, while low values (TWI < 20) are typical of steep and diverging terrains [38,39]. TWI is a proxy for the depth of the groundwater, soil pH, vegetation species richness and soil organic matter [40–42].

### 2.3. Data analysis

#### 2.3.1. Spatial autocorrelation testing

Assessment of spatial autocorrelation was performed in the preceding study [17], using Moran's *I* (MI) statistic in ArcGIS v.10. Briefly, the assessment revealed that both *Anopheles* mosquitoes (MI = 0.15) and malaria infection prevalence (MI = 0.17) were spatially auto-correlated, showing a tendency towards clustering.

#### 2.3.2. Spatial modelling and prediction

**Figure 1.**
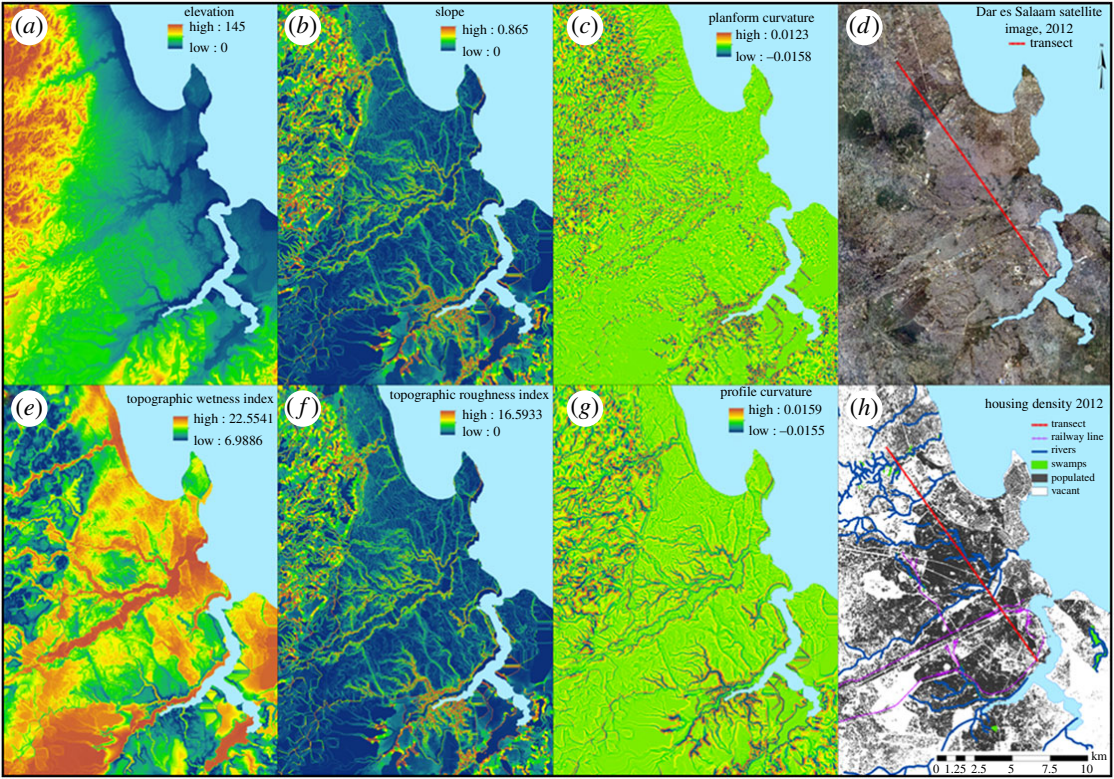
Spatial distributions and patterns of geophysical topographic predictors which significantly predicted mosquito vector densities and malaria infection prevalence.

All variables indicated in table 1 were used in the analysis. Two distinct conditional autoregressive (CAR) models adjusted for larvicide application regimes were fitted using a contiguous neighbour structured random effect to elucidate the spatial interactions that arise between geophysical topography (table 1), densities of *A. gambiae* and malaria infection prevalence and to map their spatial predictions. The CAR model accounts for spatial effects and, in essence, the mean or probability values estimated at any given location are conditional on the levels in the neighbouring areas. The model for densities of *A. gambiae* was fitted using a Poisson-lognormal CAR model [45], and malaria infection prevalence was fitted using a binomial logit CAR model [46]. The autoregression parameters and confidence intervals for each model were obtained using Markov chain Monte Carlo (MCMC) methods [46,47]. Coefficients for the *A. gambiae* model and the infection prevalence logit model were exponentiated to present relative rates and odds ratios, respectively. Significant variables are presented in figure 1. Transects of all the significant geophysical topographic variables as well as the predicted entomology/parasitology outcome in different parts of the city were prepared to aid interpretation of the results. The CAR spatial modelling was performed using CrimeStat IV® (for obtaining autoregression parameters) [48] and SAGA GIS® (for mapping the final model results), which are both open source GIS software packages. Transect values were extracted using SAGA GIS® and graphs were prepared using Microsoft Excel.

## 3. Results

### 3.1. Geophysical predictors of malaria hazard and risk at fine spatial scales

Consistent with results from previous analyses [26], the application of larvicide by means of granular *Bti*, after but not before the MoHSW took over the management of all delivery activities from an external contractor, resulted in a reduction of both densities of adult *A. gambiae* and human malaria infection prevalence (table 2). Densities of *A. gambiae* continued to decline after the MoHSW introduced the liquid larvicide formulation, and the malaria entomological inoculation rate dropped below the threshold of 0.1 infectious bites per person per year [26], below which transmission may be destabilized [50–52]. However, this had little effect upon malaria infection prevalence (table 2), presumably because of the long-term persistence of untreated, chronic malaria infections at high prevalence [26], which can take years to dissipate [53–56]. The impacts of larviciding upon vector densities estimated with these models that allow for spatial autocorrelation (table 2) were considerably greater than those previously estimated with non-spatially explicit regression models, presumably because the latter treated each surveyed location as geographically independent and did not allow for the effects of *Anopheles* mosquitoes flying into larvicide-treated areas from untreated areas nearby.

**Table 2.** Association between mosquito vector densities/malaria infection prevalence and geophysical topographic indicators of local wetness, the interface between human settlements and aquatic habitat and effective interventions. TWI: topographic wetness index; TRI: topographic roughness index; GoT granule: larviciding with granular formulation of *Bacillus thuringiensis* var. *israelensis* (*Bti*), managed by the GoT, between January and July 2011; GoT liquid: larviciding managed by the GoT using a pre-diluted liquid formulation of *Bti*, from August 2011 onwards.

	predictor	relative rate^a^ or odds ratio^b^ [95% CI]	proportion of variance^c^	relative importance^d^	*p*-value	interpretation
mean catches of adult female *Anopheles gambiae* per trap person night^a^	GoT granule	0.31 [0.14–0.69]	0.06	7.0	0.008	effective intervention
	GoT liquid	0.089 [0.087–0.092]	0.13	15.1	<0.001	effective intervention
	slope	1.78 [1.75–1.82]	0.26	30.2	0.004	wet–dry boundary
	TRI	1.11 [1.04–1.18]	0.12	14.0	0.005	wet–dry boundary
	planform curvature	0.80 [0.78–0.82]	0.29	33.7	0.001	local wetness
	adjusted *R*^2^	0.83				
	targeting ratio	85/15 (5.7 : 1)				
human malaria infection prevalence^b^	GoT granule	0.85 [0.83–0.88]^b^	0.15	25.9	<0.001	effective intervention
	TWI	1.79 [1.52–2.10]^b^	0.12	20.7	0.011	local wetness
	elevation	0.74 [0.70–0.79]^b^	0.10	17.2	0.006	local wetness
	profile curvature	3.70 [2.46–5.55]^b^	0.21	36.2	<0.001	wet–dry boundary
	adjusted *R*^2^	0.58				
	targeting ratio	60/40 (1.5 : 1)				

^a^For the Poisson-lognormal-distributed mean mosquito catch outcome variable.

^b^For the binomial-distributed human infection prevalence outcome variable.

^c^The proportion of variance is computed in two groups separately. The groups are interventions and geophysical indicators. For example, in the *A. gambiae* model, interventions contributed a total of 19% (*R*^2^ = 0.19) in the overall model. Of these, 13% is shared by GoT liquid and only 0.06% is contributed by the GoT granule. Similarly for geophysical indicators, which all together contributed a total of 67% to the model, of which planform curvature had the highest portion, which is 29%, and the smallest was contributed by TWI, which has 12%. A similar approach applies to the relative importance [49].

^d^Relative importance is the weighted average of the proportion of variance times 100% [49].

Consistent with previous reports [18–21,23,25,57–59], spatial variations of malaria vector density and human infection prevalence were associated with local geophysical topographic predictors of aquatic habitat suitability in an intuitively obvious manner (table 2). Higher densities of *A. gambiae* were found in locations with concave planform curvature (figure 1*c*) occurring at the bottoms of steep slopes, while elevated malaria infection was associated with high topographic wetness index, or with lower elevations (table 2), both of which clearly map out the bottoms of valleys and other depressions across Dar es Salaam (figure 1*e* and *a*, respectively).

However, some associations of malaria vector density and infection prevalence with additional geophysical topographic indicators were observed that were initially surprising and counterintuitive. High densities of *A. gambiae* and elevated malaria infection prevalence were associated with indicators of good natural drainage, at the boundaries between dry and wet areas (table 2), which would normally be considered least likely to provide suitable aquatic larval habitats for *Anopheles* mosquitoes [18,24,25,60–64]. Specifically, higher densities of adult *A. gambiae* mosquitoes were caught in locations with steep slopes (figure 1*b*), or with high topographic roughness index (figure 1*f*), which is essentially equivalent (table 2) to slope (figure 1*b*). Similarly, higher malaria infection prevalence among humans was surprisingly associated with convex profile curvature (figure 1*g*) occurring at the tops of steep slopes. The panels in figure 1 are grouped into vertical pairs, so that topographically similar indicators of hydrology and ecosystem geography can be compared and contrasted: while figure 1*a*,*e* clearly map out valleys and depressions with high local water accumulation and wetness, figure 1*b*,*f* clearly delineate the well-drained slopes leading from the bottoms of those wet valleys to the higher, drier areas above them where most of the residents of Dar es Salaam actually live. In figure 1*c*,*g* it can be seen that these two geophysical topographic indicators map out the tops of those slopes at the edges of valleys.

**Figure 2.**
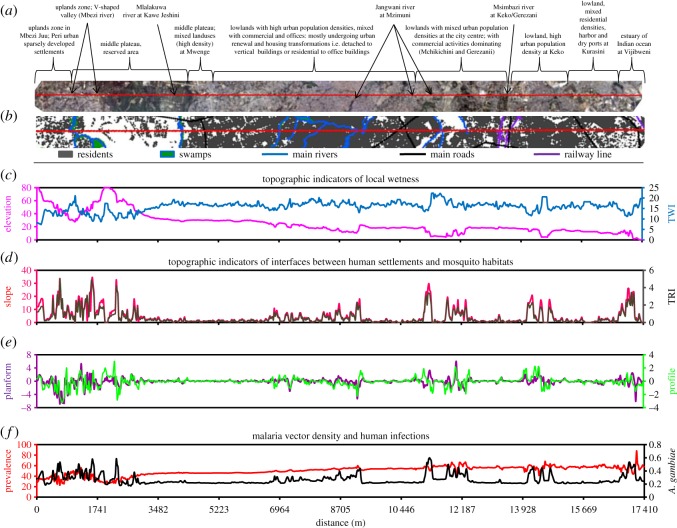
Profile of the transect illustrating the cross-cutting relationship between geophysical topography and human population density as well as the entomological and epidemiological indicators.

To illustrate how these three different sets of geophysical topographic indicators of elevated malaria hazard and risk are closely associated with each other, and often vary across their full range of values within distances of less than 100 m, a detailed geophysical topographic, entomological and epidemiological profile of the transect illustrated in figure 1*d* is presented in figure 2. Figure 2 illustrates how steep slopes, high topographic roughness index, high (convex) profile curvature and high (convex) planform curvature are found at the edges of valleys with low elevation and high topographic wetness index. The most obvious biological explanation for this observation is that these interfaces between lowlands and uplands are where most host-seeking adult *Anopheles* mosquitoes first encounter and attack humans, when they disperse in search of blood after emerging or ovipositing.

**Figure 3.**
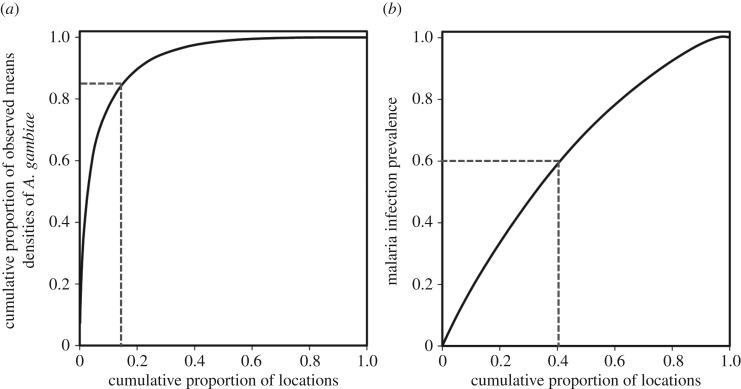
(*a*,*b*) Predicted spatial distribution of locations with the highest mosquito vector densities and malaria infection prevalence.

The model fits as measured by adjusted *R*^2^ were 0.86 in the *A. gambiae* density model and 0.58 in the malaria infection model. Compared with previous analyses [26], models combining geophysical topographic predictors with larvicide intervention status account for very high proportions of variance in *A. gambiae* densities but far less so for malaria infection prevalence. Only three geophysical topographic predictors of *A. gambiae* accounted for more than half (67%) of the variance in malaria vector density transmission hazard, with most of the remainder (19%) accounted for by the larvicide application status (table 2). By contrast, the geophysical topographic indicators of malaria prevalence accounted for only 43% of variance in this indicator of infection burden (table 2). Indeed, the map of the vector densities predicted by the fitted model presented in table 2 (figure 3*a*) has far greater contrast than that for predicted malaria infection prevalence (figure 3*b*). Vector densities are predicted to be very focal; indeed, they are specifically localized along the edges of valleys so that the cartographic expression of this model fit (figure 3*a*) is essentially identical to maps of geophysical topographic variables capturing steep slopes (figure 1*b* and *f*) or convex curvatures at the tops of those steep slopes (figure 1*c* and *g*). By contrast, malaria infection prevalence among humans is far more homogeneously distributed across the city, with what little contrast there is in the maps of predicted risk (figure 3*b*) defining the flood-prone valley bottoms where very few people actually live.

**Figure 4.**
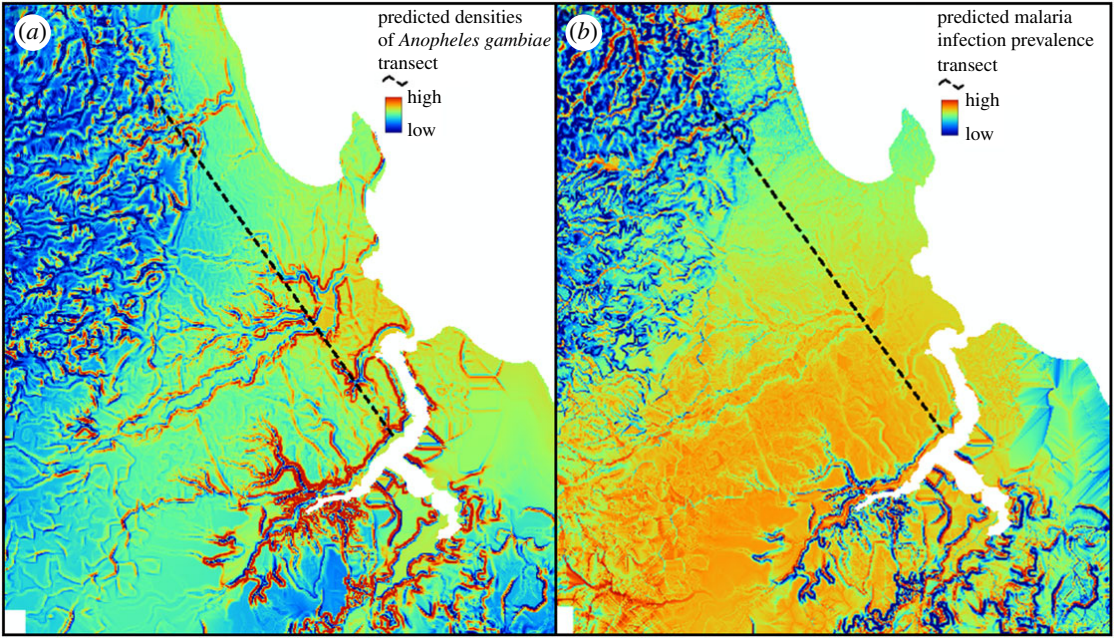
(*a*,*b*) Spatial targeting efficiency of the locations with the highest predicted densities of *A. gambiae* and human infection prevalence.

Correspondingly, geophysical topographic indicators provide a far more selective basis for geographically targeting the vectors of malaria (figure 4*a*) than human infections (figure 4*b*) in Dar es Salaam. Figure 4*a* illustrates just how selective targeting of adult vector control to these geophysical topographically mapped boundaries could be: geographical targeting in order of model-predicted vector density would account for 85% of all the *A. gambiae* caught if only the 15% of survey locations with the highest predicted hazard were included. By contrast, figure 4*b* reveals that only 60% of detected malaria infections would be captured by targeting a full 40% of survey locations with the highest predicted infection prevalence risk. Expressing these proportions of locations and mosquitoes or malaria infections covered relative to each other gives estimated targeting ratios of 5.7 and 1.5 to 1, respectively (figure 4).

## 4. Discussion

The results of this study illustrate, for the first time, that while conventional geophysical topographic predictors of wetness, such as concave planform curvature, topographic wetness index and low elevation, may be valuable for spatial targeting of larval source management, indicators of dryness on steep slopes, and at the tops of those steep slopes, also have unforeseen utility for targeting adult malaria vector mosquitoes. These steep, dry slopes occur at the periphery of human settlements, immediately adjacent to wet, low-lying areas with abundant aquatic habitat. This is therefore where residents are disproportionately exposed to adult *Anopheles* mosquitoes dispersing from nearby aquatic habitats after emerging or ovipositing [2–4,8,65].

The observed predictive value of geophysical topographic indicators of wetness for elevated densities of *A. gambiae* and malaria infection prevalence, predominantly in the main river valleys, drainage lines and local depressions of the city landscape [44], is fully consistent with previous studies in Kenya [18,63,66,67] and Tanzania [19,68,69]. However, the observation that local geophysical topography predicts not only the distribution of aquatic habitats but also the peripheries of human settlements that surround them is novel and was not foreseen at the outset of the study. These narrow boundaries between dry human habitat and wet mosquito habitat are where malaria vectors first encounter and attack human hosts when they seek blood after emerging or ovipositing. They could therefore be exploited to define target zones for *barrier* interventions to kill adult mosquitoes, such as the residual surface spraying [70] methods this term was originally applied to [71], or alternatives such as insecticide netting barriers [72], vapour-phase insecticide emanators [73] or nocturnal space spraying [74]. Barrier targeting of adult vector control at high spatial resolution (figure 4*a*) would not only directly protect the local residents but also shield the rest of the population in upland areas further away from these wet valleys [70,75], thus maximizing communal benefit to all residents through mass suppression of vector populations.

Similar geophysical topographic indicators of abundant vector larval habitat, and the steep banks of the valleys, where they usually occur, were also predictive of human infection prevalence but had far less predictive power. The weaker predictive value may well be explained by variations in human resilience factors, such window screening, bed net use and time spent indoors [26], all of which could attenuate the dependence of infection risk on exposure hazard. For example, human infection prevalence is known to be highly dependent on local vector density among households lacking window screens but is essentially unaffected by it in well-screened houses [26]. Furthermore, many of the human infections detected in these surveys appear to have been long-standing chronic infections [26] that might not necessarily be directly related to ongoing transmission exposure. Also, what little contrast can be seen in maps of model-predicted malaria infection risk depicts the wet valley bottoms (figure 4*b*) where very few people actually live (figures 1*h* and 2*b*). Such geophysical topographic indicators of likely infection burden are therefore far less useful for targeting interventions to increase human resilience against exposure hazard, such as bed nets, mosquito-proofed housing or repellents, as well as testing and treatment or mass administration of curative drugs to tackle chronic morbidity burden [76] and clear the infectious reservoir [77], than they are for targeting vector population suppression interventions against adult vectors.

The approach presented could be readily applied and assessed in other African settings, as a means to identify heterogeneity in malaria risk patterns and detect zones for spatially selective targeted interventions. Specifically, it may be used to predict the distribution of malaria transmission across the landscapes where dense human populations surround aquatic habitats such as rivers, swamps and flooding zones, or vice versa in some flood-prone rural settings. In urban Africa, these zones tend to be illegally occupied informal settlements [78], inhabited mostly by the poor whose human resilience against malaria risk is very low [79,80]. To tap the full potential of this approach to mapping areas for selectively targeted interventions against adult mosquitoes, the resolution of geophysical indicators needs to be high enough to accurately capture transmission at fine spatial scales that hazard and risk really occur at [17,81]. For this reason, the approach is really only suitable for use by locally managed, decentralized mosquito control programmes that can practically target appropriate interventions at very fine geographical scales, rather than for cruder prioritization of interventions across larger provincial or national scales [20,82,83]. Beyond evaluating in a wider diversity of settings where sufficient entomological and epidemiological data are available that have been mapped at high resolution, this approach also needs to be field-validated by empirically evaluating the actual, rather than the predicted, impacts of vector control activities directed by these targeting criteria.

Another limitation of this study is that these spatial models relied exclusively on geophysical topographical factors as the only geographical predictor variables. The formation of larval *A. gambiae* habitats and risk of malaria transmission also depend on many other geographically variable factors [84,85], such as ground water levels, soil characteristics, weather, vegetation, human behaviour, land use, drainage infrastructure and education, as well as health service access and quality [86,87]. It is therefore possible that better predictive models of malaria infection risk could be developed using richer sets of independent indicator variables.

## 5. Conclusion

Geophysical topographic indicators of local water accumulation potential can be used to target not only aquatic habitats with larval source management but also the peripheries of human settlements along the drier banks of these wet valleys, where adult *Anopheles* mosquitoes seeking blood first encounter and attack the residents. Vector control interventions against adult mosquitoes, such as residual surface spraying [70,71], treated netting barriers [72], vapour-phase insecticides [73] or nocturnal space sprays [74], could be selectively and efficiently deployed to barrier zones using geophysical topographic predictors of steep slopes and the tops of those steep slopes.

## Data accessibility

Our data are available in Dryad Digital Repository [88].

## References

[1] Bousema T et al. 2016 The impact of hotspot-targeted interventions on malaria transmission in Rachuonyo south district in the western Kenyan highlands: a cluster-randomized controlled trial. PLoS Med. 13, e1001993 (doi:10.1371/journal.pmed.1001993) 27071072 10.1371/journal.pmed.1001993 PMC4829260

[2] Keiser J, Utzinger J, Castro MC, Smith TA, Tanner T, Singer BH 2004 Urbanization in sub-Saharan Africa and implication for malaria control. Am. J. Trop. Med. Hyg. 71, 118–127. 15331827

[3] Killeen GF, Knols BG, Gu W 2003 Taking malaria transmission out of the bottle: implications of mosquito dispersal for vector-control interventions. Lancet Infect. Dis. 3, 297–303. (doi:10.1016/S1473-3099(03)00611-X) 12726980 10.1016/s1473-3099(03)00611-x

[4] Menach A, McKenzie FE, Flahault A, Smith D 2005 The unexpected importance of mosquito oviposition behaviour for malaria: non-productive larval habitats can be sources for malaria transmission. Malar. J. 4, 23 (doi:10.1186/1475-2875-4-23) 15892886 10.1186/1475-2875-4-23 PMC1164431

[5] Killeen GF, McKenzie FE, Foy BD, Schieffelin C, Billingsley PF, Beier JC 2000 A simplified model for predicting malaria entomologic inoculation rates based on entomologic and parasitologic parameters relevant to control. Am. J. Trop. Med. Hyg. 62, 535–544. (doi:10.4269/ajtmh.2000.62.535) 11289661 10.4269/ajtmh.2000.62.535 PMC2483339

[6] Smith DL, Mckenzie FE 2004 Statics and dynamics of malaria infection in *Anopheles* mosquitoes. Malar. J. 3, 13 (doi:10.1186/1475-2875-3-13) 15180900 10.1186/1475-2875-3-13 PMC449722

[7] Keiser J, Singer BH, Utzinger J 2005 Reducing the burden of malaria in different eco-epidemiological settings with environmental management: a systematic review. Lancet Infect. Dis. 5, 695–708. (doi:10.1016/S1473-3099(05)70268-1) 16253887 10.1016/S1473-3099(05)70268-1

[8] Robert V, MacIntyre K, Keating J, Trape JF, Duchemin JB, Warren M, Beier JC 2003 Malaria transmission in urban sub-Saharan Africa. Am. J. Trop. Med. Hyg. 68, 169–176. 12641407

[9] Fillinger U, Lindsay SW 2011 Larval source management for malaria control in Africa: myths and reality. Malar. J. 10, 353 (doi:10.1186/1475-2875-10-353) 22166144 10.1186/1475-2875-10-353 PMC3273449

[10] Hay SI, Guerra CA, Tatem AJ, Atkinson PM, Snow RW 2005 Urbanization, malaria transmission and disease burden in Africa. Nat. Rev. Microbiol. 3, 81–90. (doi:10.1038/nrmicro1069) 15608702 10.1038/nrmicro1069 PMC3130901

[11] WHO. 2013 Larval source management—a supplementary measure for malaria vector control: an operational manual. Geneva, Switzerland: World Health Organization.

[12] Bousema T, Griffin JT, Sauerwein RW, Smith DL, Churcher TS, Takken W, Ghani A, Drakeley C, Gosling R 2012 Hitting hotspots: spatial targeting of malaria for control and elimination. PLoS Med. 9, e1001165 (doi:10.1371/journal.pmed.1001165) 22303287 10.1371/journal.pmed.1001165 PMC3269430

[13] Bejon P et al. 2010 Stable and unstable malaria hotspots in longitudinal cohort studies in Kenya. PLoS Med. 7, e1000304 (doi:10.1371/journal.pmed.1000304) 20625549 10.1371/journal.pmed.1000304 PMC2897769

[14] Bejon P et al. 2014 A micro-epidemiological analysis of febrile malaria in Coastal Kenya showing hotspots within hotspots. eLife 3, e02130 (doi:10.7554/eLife.02130) 24843017 10.7554/eLife.02130 PMC3999589

[15] Gaudart J et al. 2006 Space-time clustering of childhood malaria at the household level: a dynamic cohort in a Mali village. BMC Public Health 6, 1–13. (doi:10.1186/1471-2458-6-286) 17118176 10.1186/1471-2458-6-286 PMC1684261

[16] Carter R, Mendis KN, Roberts DR 2000 Spatial targeting of interventions against malaria. Bull. World Health Organ. 78, 1401–1411. 11196487 PMC2560653

[17] Mwakalinga VM et al. 2016 Spatially aggregated clusters and scattered smaller loci of elevated malaria vector density and human infection prevalence in urban Dar es Salaam, Tanzania. Malar. J. 15, 135 (doi:10.1186/s12936-016-1186-9) 26931372 10.1186/s12936-016-1186-9 PMC4774196

[18] Atieli HE, Zhou G, Lee MC, Kweka EJ, Afrane Y, Mwanzo I, Githeko AK, Yan G 2011 Topography as a modifier of breeding habitats and concurrent vulnerability to malaria risk in the western Kenya highlands. Parasit. Vectors 4, 241 (doi:10.1186/1756-3305-4-241) 22196078 10.1186/1756-3305-4-241 PMC3269397

[19] Balls MJ, Bodker R, Thomas CJ, Kisinza W, Msangeni HA, Lindsay SW 2004 Effect of topography on the risk of malaria infection in the Usambara Mountains, Tanzania. Trans. R. Soc. Trop. Med. Hyg. 98, 400–408. (doi:10.1016/j.trstmh.2003.11.005) 15138076 10.1016/j.trstmh.2003.11.005

[20] Cohen JM, Ernst KC, Lindblade KA, Vulule JM, John CC, Wilson ML 2010 Local topographic wetness indices predict household malaria risk better than land-use and land-cover in the western Kenya highlands. Malar. J. 9, 1475–2875. 10.1186/1475-2875-9-328 PMC2993734 21080943

[21] Cohen JM, Ernst KC, Lindblade KA, Vulule JM, John CC, Wilson ML 2008 Topography-derived wetness indices are associated with household-level malaria risk in two communities in the western Kenyan highlands. Malar. J. 7, 40 (doi:10.1186/1475-2875-7-40) 18312633 10.1186/1475-2875-7-40 PMC2276221

[22] Mushinzimana E et al. 2006 Landscape determinants and remote sensing of anopheline mosquito larval habitats in the western Kenya highlands. Malar. J. 5, 13 (doi:10.1186/1475-2875-5-13) 16480523 10.1186/1475-2875-5-13 PMC1420309

[23] Githeko AK, Ayisi JM, Odada PK, Atieli FK, Ndenga BA, Githure JI, Yan G 2006 Topography and malaria transmission heterogeneity in western Kenya highlands: prospects for focal vector control. Malar. J. 5, 107 (doi:10.1186/1475-2875-5-107) 17096835 10.1186/1475-2875-5-107 PMC1654174

[24] Smith MW, Macklin MG, Thomas CJ 2013 Hydrological and geomorphological controls of malaria transmission. Earth Sci. Rev. 116, 109–127. (doi:10.1016/j.earscirev.2012.11.004)

[25] Nmor J, Sunahara T, Goto K, Futami K, Sonye G, Akweywa P, Dida G, Minakawa N 2013 Topographic models for predicting malaria vector breeding habitats: potential tools for vector control managers. Parasit. Vectors 6, 14 (doi:10.1186/1756-3305-6-14) 23324389 10.1186/1756-3305-6-14 PMC3617103

[26] Msellemu D et al. 2016 The epidemiology of residual *Plasmodium falciparum* malaria transmission and infection burden in an African city with high coverage of multiple vector control measures. Malar. J. 15, 288 (doi:10.1186/s12936-016-1340-4) 27216734 10.1186/s12936-016-1340-4 PMC4877954

[27] Chaki P, Kannady K, Mtasiwa D, Tanner M, Mshinda H, Kelly AH, Killeen GF 2014 Institutional evolution of a community-based programme for malaria control through larval source management in Dar es Salaam, United Republic of Tanzania: a case study. Malar. J. 13, 245 (doi:10.1186/1475-2875-13-245) 24964790 10.1186/1475-2875-13-245 PMC4082415

[28] Fillinger U et al. 2008 A tool box for operational mosquito larval control: preliminary results and early lessons from the urban malaria control programme in Dar es Salaam, Tanzania. Malar. J. 7, 20 (doi:10.1186/1475-2875-7-20) 18218148 10.1186/1475-2875-7-20 PMC2259364

[29] NBS RCO 2014 Dar es Salaam region socio-economic profile. Dar es Salaam, The United Republic of Tanzania: Prime Minister's Office Regional Administration and Local Government.

[30] Dongus S, Mwakalinga V, Kannady K, Tanner M, Killeen G 2011 Participatory mapping as a component of operational malaria vector control in Tanzania: geospatial analysis of environmental health. In Geotechnologies and the environment, vol. 4 (eds Maantay JA, McLafferty S), pp. 321–336. Amsterdam, The Netherlands: Springer.

[31] Detto M, Muller-Landau HC, Mascaro J, Asner GP 2013 Hydrological networks and associated topographic variation as templates for the spatial organization of tropical forest vegetation. PLoS ONE 8, e76296 (doi:10.1371/journal.pone.0076296) 24204610 10.1371/journal.pone.0076296 PMC3799763

[32] Ohlmacher GC 2007 Plan curvature and landslide probability in regions dominated by earth flows and earth slides. Eng. Geol. 91, 117–134. (doi:10.1016/j.enggeo.2007.01.005)

[33] Warren SD, Hohmann MG, Auerswald K, Mitasova H 2004 An evaluation of methods to determine slope using digital elevation data. CATENA 58, 215–233. (doi:10.1016/j.catena.2004.05.001)

[34] Weiss A 2001 Topographic position and landforms analysis. See http://www.jennessent.com/downloads/tpi-poster-tnc_18x22.pdf.

[35] Riley SJ, DeGloria SD, Elliot R 1999 A terrain ruggedness index that quantifies topographic heterogeneity. Int. J. Sci. 5, 1–4.

[36] Tarboton DG 1997 A new method for the determination of flow directions and contributing areas in grid digital elevation models. Water Resour. 33, 309–319. (doi:10.1029/96WR03137)

[37] Moore ID, Grayson RB, Ladson AR 1991 Digital terrain modeling—a review of hydrological, geomorphological, and biological applications. Hydrol. Processes 5, 3–30. (doi:10.1002/hyp.3360050103)

[38] Schmidt F, Persson A 2003 Comparison of DEM data capture and topographic wetness indices. Prec. Agric. 4, 179–192. (doi:10.1023/A:1024509322709)

[39] Beven KJ, Kirkby MJ 1979 A physically based, variable contributing area model of basin hydrology. Hydrol. Sci. Bull. 24, 43–69. (doi:10.1080/02626667909491834)

[40] Pei T, Qin C-Z, Zhu AX, Yang L, Luo M, Li B, Zhou C 2010 Mapping soil organic matter using the topographic wetness index: a comparative study based on different flow-direction algorithms and Kriging methods. Ecol. Indic. 10, 610–619. (doi:10.1016/j.ecolind.2009.10.005)

[41] Sorensen R, Zinko U, Seibert J 2006 On calculation of the topographic wetness index: evaluation of different methods based on field observations. Hydrol. Earth Sys. Sci. Discuss. 10, 1807–1834.

[42] Grabs T, Seibert J, Bishop K, Laudon H 2009 Modeling spatial patterns of saturated areas: a comparison of the topographic wetness index and a dynamic distributed model. J. Hydrol. 373, 15–23. (doi:10.1016/j.jhydrol.2009.03.031)

[43] Böhner J, McCloy KR, Strobl J 2014 SAGA — analysis and modelling applications. Göttingen, Germany: Göttinger Geographische Abhandlungen.

[44] Dongus S et al. 2007 Participatory mapping of target areas to enable routine comprehensive larviciding of malaria vector mosquitoes in Dar es Salaam, Tanzania. Int. J. Health Geogr. 6, 37 (doi:10.1186/1476-072X-6-37) 17784963 10.1186/1476-072X-6-37 PMC2025588

[45] Wany Y, Kockelman KM 2013 A Poisson-lognormal conditional-autoregressive model for multivariate spatial analysis of pedestrian crash counts across neighborhoods. Accident Anal. Prev. 60, 71–84. (doi:10.1016/j.aap.2013.07.030) 10.1016/j.aap.2013.07.030 24036167

[46] Gelfand AE, Vounatsou P 2003 Proper multivariate conditional autoregressive models for spatial data analysis. Biostatistics 4, 11–25. (doi:10.1093/biostatistics/4.1.11) 12925327 10.1093/biostatistics/4.1.11

[47] Waller LA, Gotway CA 2004 Applied spatial statistics for public health data. Hoboken, NJ: John Wiley & Sons.

[48] Levine N 2013 Crimestat IV: a spatial statistics program for the analysis of crime incident locations, version 4.0, vol. IV Washington, DC: The U.S. Department of Justice.

[49] Aslibekyan S, Wiener HW, Wu G, Zhi D, Shrestha S, de los Campos G, Vazquez A 2014 Estimating proportions of explained variance: a comparison of whole genome subsets. BMC Proc. 8, (Suppl. 1), S102–S102. 25519356 10.1186/1753-6561-8-S1-S102 PMC4143698

[50] Beier JC, Killeen GF, Githure J 1999 Short report: entomologic inoculation rates and *Plasmodium falciparum* malaria prevalence in Africa. Am. J. Top. Med. Hyg. 61, 109–113. (doi:10.4269/ajtmh.1999.61.109) 10.4269/ajtmh.1999.61.109 10432066

[51] Smith DL, Dushoff J, Snow RW, Hay SI 2005 The entomological inoculation rate and *Plasmodium falciparum* infection in African children. Nature 438, 492–495. (doi:10.1038/nature04024) 16306991 10.1038/nature04024 PMC3128496

[52] Smith DL, McKenzie FE, Snow RW, Hay SI 2007 Revisiting the basic reproductive number for malaria and its implications for malaria control. PLoS Biol. 5, e42 (doi:10.1371/journal.pbio.0050042) 17311470 10.1371/journal.pbio.0050042 PMC1802755

[53] Bretscher MT, Maire N, Chitnis N, Felger I, Owusu-Agyei S, Smith T 2011 The distribution of *Plasmodium falciparum* infection durations. Epidemics 3, 109–118. (doi:10.1016/j.epidem.2011.03.002) 21624782 10.1016/j.epidem.2011.03.002

[54] Sama W, Killeen GF, Smith T 2004 Estimating the duration of *Plasmodium falciparum* infection from malaria eradication trials. Am. J. Top. Med. Hyg. 70, 625–634. 15211003

[55] Ashley EA, White NJ 2014 The duration of *Plasmodium falciparum* infections. Malar. J. 13, 1–11. (doi:10.1186/1475-2875-13-1) 25515943 10.1186/1475-2875-13-500 PMC4301960

[56] Smith DL, Hay SI 2009 Endemicity response timelines for *Plasmodium falciparum* elimination. Malar. J. 8, 87 (doi:10.1186/1475-2875-8-87) 19405974 10.1186/1475-2875-8-87 PMC2686731

[57] Asefi S, Li J, Nair US, Ray DK, Welch RM, Padilla N, Barrios E, Benedict ME 2005 An integrated hydrological and atmospheric model to predict malaria epidemics In *Proc. 21st Int. Conf. on Interactive Information and Processing Systems* (*IIPS*) *for Meteorology, Oceanography, and Hydrology*, J9.4. San Diego, CA, American Meteorological Society.

[58] Bomblies A, Duchemin JB, Eltahir EAB 2008 Hydrology of malaria: model development and application to a Sahelian village. Water Resour. 44, W12445.

[59] Soleimani-Ahmadi M, Vatandoost H, Zare M, Turki H, Alizadeh A 2015 Topographical distribution of anopheline mosquitoes in an area under elimination programme in the south of Iran. Malar. J. 14, 1–8. (doi:10.1186/s12936-015-0771-7) 26148647 10.1186/s12936-015-0771-7 PMC4491864

[60] McCann RS, Messina JP, MacFarlane DW, Bayoh MN, Vulule JM, Gimnig JE, Walker ED 2014 Modeling larval malaria vector habitat locations using landscape features and cumulative precipitation measures. Int. J. Health Geogr. 13, 1–12. (doi:10.1186/1476-072X-13-17) 24903736 10.1186/1476-072X-13-17 PMC4070353

[61] Ndoen E, Wild C, Dale P, Sipe N, Dale M 2010 Relationships between anopheline mosquitoes and topography in West Timor and Java, Indonesia. Malar. J. 9, 242 (doi:10.1186/1475-2875-9-242) 20796265 10.1186/1475-2875-9-242 PMC2939620

[62] Hardy AJ, Gamarra JGP, Cross DE, Macklin MG, Smith MW, Kihonda J, Killeen GF, Ling'ala GN, Thomas CJ 2013 Habitat hydrology and geomorphology control the distribution of malaria vector larvae in rural Africa. PLoS ONE 8, e81931 (doi:10.1371/journal.pone.0081931) 24312606 10.1371/journal.pone.0081931 PMC3849348

[63] Minakawa N, Munga S, Atieli F, Mushinzimana E, Zhou G, Githeko AK, Yan G 2005 Spatial distribution of anopheline larval habitats in Western Kenyan highlands: effects of land cover types and topography. Am. J. Trop. Med. Hyg. 73, 157–165. 16014851

[64] Chikodzi D 2013 Spatial modelling of malaria risk zones using environmental, anthropogenic variables and geographical information systems techniques. J. Geosci. Geomatics 1, 8–14.

[65] Smith DL, Dushoff J, McKenzie FE 2004 The risk of a mosquito-borne infection in a heterogeneous environment. PLoS Biol. 2, e368 (doi:10.1371/journal.pbio.0020368) 15510228 10.1371/journal.pbio.0020368 PMC524252

[66] Minakawa N, Mutero CM, Githure JI, Beier JC, Yan G 1999 Spatial distribution and habitat characterization of anopheline mosquito larvae in Western Kenya. Am. J. Trop. Med. Hyg. 61, 1010–1016. (doi:10.4269/ajtmh.1999.61.1010) 10674687 10.4269/ajtmh.1999.61.1010

[67] Munga S, Minakawa N, Zhou G, Mushinzimana E, Barrack OO, Githeko AK, Yan G 2006 Association between land cover and habitat productivity of malaria vectors in western Kenyan highlands. Am. J. Trop. Med. Hyg. 74, 69–75. 16407348

[68] Mboera LEG, Kamugisha ML, Malima RC, Mushi AK, Msuya FH, Massawe T, Kitua AY 2002 Malaria prevalence and health-seeking behaviours among communities of the lowlands and highlands of Gonja, Same District, Tanzania. Tanzan. Health Res. Bull. 4, 47–53.

[69] Mboera LE, Kamugisha ML, Rumisha SF, Kisinza WN, Senkoro KP, Kitua AY 2008 Malaria and mosquito net utilisation among schoolchildren in villages with or without healthcare facilities at different altitudes in Iringa District, Tanzania. Afr. Health Sci. 8, 114–119. 19357761 PMC2584322

[70] Perich MJ, Tidwell MA, Dobson SE, Sardelis MR, Zaglul A, Williams DC 1993 Barrier spraying to control the malaria vector *Anopheles albimanus*: laboratory and field evaluation in the Dominican Republic. Med. Vet. Entomol. 7, 363–368. (doi:10.1111/j.1365-2915.1993.tb00706.x) 8268492 10.1111/j.1365-2915.1993.tb00706.x

[71] Fay RW, Kilpatrick JW 1958 Insecticides for control of adult diptera. Annu. Rev. Entomol. 3, 401–420. (doi:10.1146/annurev.en.03.010158.002153)

[72] Faiman R, Warburg A 2012 Insecticide-treated vertical mesh barriers reduce the number of biting mosquitoes. Med. Vet. Entomol. 26, 26–32. (doi:10.1111/j.1365-2915.2011.00966.x) 21615442 10.1111/j.1365-2915.2011.00966.x

[73] Govella NJ, Ogoma SB, Paliga J, Chaki PP, Killeen GF 2015 Impregnating hessian strips with the repellent pyrethroid transfluthrin prevents outdoor exposure to vectors of malaria and lymphatic filariasis in urban Dar es Salaam, Tanzania. Parasit. Vectors 8, 322 (doi:10.1186/s13071-015-0937-8) 26063216 10.1186/s13071-015-0937-8 PMC4465323

[74] Bonds JA 2012 Ultra-low-volume space sprays in mosquito control: a critical review. Med. Vet. Entomol. 26, 121–130. (doi:10.1111/j.1365-2915.2011.00992.x) 22235908 10.1111/j.1365-2915.2011.00992.x

[75] Charlwood JD et al. 1998 Cordon sanitaire or laissez faire: differential dispersal of young and old females of the malaria vector Anopheles funestus Giles (Diptera: Culicidae) in southern Mozambique. African Entomol. 6, 1–6.

[76] Chen I, Clarke SE, Gosling R, Hamainza B, Killeen G, Magill A, O'Meara WP, Price RN, Riley EM 2016 ‘Asymptomatic’ malaria: a chronic and debilitating infection that should be treated. PLoS Med. 13, e1001942 (doi:10.1371/journal.pmed.1001942) 26783752 10.1371/journal.pmed.1001942 PMC4718522

[77] Bousema T, Okell L, Felger I, Drakeley C 2014 Asymptomatic malaria infections: detectability, transmissibility and public health relevance. Nat. Rev. Microbiol. 12, 833–840. (doi:10.1038/nrmicro3364) 25329408 10.1038/nrmicro3364

[78] Sakijege T, Lupala J, Sheuya S 2012 Flooding, flood risks and coping strategies in urban informal residential areas: the case of Keko Machungwa, Dar es Salaam, Tanzania. Jàmbá: J. Disaster Risk Studies 4, a46 (doi:10.4102/jamba.v4i1.46)

[79] Salami RO, von Meding JK, Giggins H 2017 Urban settlements' vulnerability to flood risks in African cities: a conceptual framework. Jàmbá: J. Disaster Risk Studies 9, a370. 10.4102/jamba.v9i1.370 PMC6014121 29955335

[80] Ramin B 2009 Slums, climate change and human health in sub-Saharan Africa. Bull. World Health Organ. 87, 886 (doi:10.2471/BLT.09.073445) 20454473 10.2471/BLT.09.073445 PMC2789375

[81] Mlacha YP et al. 2017 Fine scale mapping of malaria infection clusters by using routinely collected health facility data in urban Dar es Salaam, Tanzania. Geospat. Health 12, 494 (doi:10.4081/gh.2017.494) 28555474 10.4081/gh.2017.494

[82] Hardy A et al. 2015 Mapping hotspots of malaria transmission from pre-existing hydrology, geology and geomorphology data in the pre-elimination context of Zanzibar, United Republic of Tanzania. Parasit. Vectors 8, 1–15. (doi:10.1186/s13071-015-0652-5) 25608875 10.1186/s13071-015-0652-5 PMC4307680

[83] Midega JT et al. 2012 Wind direction and proximity to larval sites determines malaria risk in Kilifi District in Kenya. Nat. Commun. 3, 674 (doi:10.1038/ncomms1672) 22334077 10.1038/ncomms1672 PMC3292715

[84] Leonardo LR, Rivera PT, Crisostomo BA, Sarol JN, Bantayan NC, Tiu WU, Bergquist NR 2005 A study of the environmental determinants of malaria and schistosomiasis in the Philippines using remote sensing and geographic information systems. Parassitologia 47, 105–114. 16044679

[85] Donnelly MJ, Birley MH, Konradsen F 1997 *An investigation of the relationship between depth to groundwater and malaria prevalence, Punjab Pakistan*, vol. 10. IIMI working paper no. 40. Colombo, Sri Lanka: International Irrigation Management Institute. 10.1080/00034989760770 9425370

[86] Bates I, Fenton C, Gruber J, Lalloo D, Lara AM, Squire SB, Theobald S, Thomson R, Tolhurst R 2004 Vulnerability to malaria, tuberculosis, and HIV/AIDS infection and disease. Part 1: determinants operating at individual and household level. Lancet Infect. Dis. 4, 267–277. (doi:10.1016/S1473-3099(04)01002-3) 15120343 10.1016/S1473-3099(04)01002-3

[87] Castro MC, Yamagata Y, Mtasiwa D, Tanner M, Utzinger J, Keiser J, Singer BH 2004 Integrated urban malaria control: a case study in Dar es Salaam, Tanzania. Am. J. Trop. Med. Hyg. 71, 103–117. 15331826

[88] Mwakalinga VM et al. 2018 Data from: Topographic mapping of the interfaces between human and aquatic mosquito habitats to enable barrier targeting of nterventions against malaria vectors Dryad Digital Repository. (doi:10.5061/dryad.77vq6gs) 10.1098/rsos.161055 PMC5990771 29892341

